# Tumor and Cerebrospinal Fluid microRNAs in Primary Central Nervous System Lymphomas

**DOI:** 10.3390/cancers11111647

**Published:** 2019-10-25

**Authors:** Michalina Zajdel, Grzegorz Rymkiewicz, Maria Sromek, Maria Cieslikowska, Pawel Swoboda, Mariusz Kulinczak, Krzysztof Goryca, Zbigniew Bystydzienski, Katarzyna Blachnio, Beata Ostrowska, Anita Borysiuk, Agnieszka Druzd-Sitek, Jan Walewski, Magdalena Chechlinska, Jan Konrad Siwicki

**Affiliations:** 1 Department of Immunology, Maria Sklodowska-Curie Institute—Oncology Center, 02-781 Warsaw, Poland; Michalina.Zajdel@coi.pl (M.Z.);; 2 Flow Cytometry Laboratory, Department of Pathology and Laboratory Diagnostics, Maria Sklodowska-Curie Institute—Oncology Center, 02-781 Warsaw, Poland; Grzegorz.Rymkiewicz@coi.pl (G.R.);; 3 Department of Medical Genetics, Maria Sklodowska-Curie Institute—Oncology Center, 02-781 Warsaw, Poland; 4 Core Facilities CeNT, University of Warsaw, 02-097 Warsaw, Poland; 5 Department of Lymphoid Malignancies, Maria Sklodowska-Curie Institute—Oncology Center, 02-781 Warsaw, Poland

**Keywords:** central nervous system (CNS) lymphoma, microRNA, brain stereotactic biopsy, CNS disease, neurological disease, cerebrospinal fluid, differential diagnosis, brain tumor

## Abstract

Primary central nervous system lymphoma (PCNSL) is a rare, highly aggressive, extranodal form of non-Hodgkin lymphoma, predominantly diagnosed as primary diffuse large B-cell lymphoma of the central nervous system (CNS DLBCL). Fast and precise diagnosis of PCNSL is critical yet challenging. microRNAs, important regulators in physiology and pathology are potential biomarkers. In 131 patients with CNS DLBCL and with non-malignant brain lesions (n-ML), miR-21, miR-19b and miR-92a, miR-155, miR-196b, miR-let-7b, miR-125b, and miR-9 were examined by RT-qPCR in brain biopsy samples (formalin-fixed paraffin-embedded tissues, FFPET; CNS DLBCL, *n* = 52; n-ML, *n* = 42) and cerebrospinal fluid samples (CSF; CNS DLBCL, *n* = 30; n-ML, *n* = 23) taken for routine diagnosis. FFPET samples were split into study and validation sets. Significantly higher CSF levels of miR-21, miR-19b, and miR-92a were identified in PCNSL but not in n-ML, and differentiated PCNSL from n-ML with 63.33% sensitivity and 80.77% specificity. In FFPETs, miR-155 and miR-196b were significantly overexpressed and miR-let-7b, miR-125b, and miR-9 were downregulated in PCNSL as compared to n-ML. Combined miR-155 and miR-let-7b expression levels in FFPETs discriminated PCNSL and n-ML with a 97% accuracy. In conclusion, tissue miR-155, miR-196b, miR-9, miR-125b, and miR-let-7b expression profiles differentiate PCNSL from n-ML. PCNSL CSFs and the relevant biopsy samples are characterized by specific, different microRNA profiles. A logistic regression model is proposed to discriminate between PCNSL and non-malignant brain lesions. None of the examined microRNAs influenced overall survival of PCNSL patients. Further ongoing developments involve next generation sequencing-based profiling of biopsy and CSF samples.

## 1. Introduction

Primary central nervous system lymphoma (PCNSL) is a highly aggressive, extranodal form of non-Hodgkin lymphoma, predominantly of the primary diffuse large B-cell lymphoma of the central nervous system (CNS DLBCL) [1,2]. PCNSL represents all primary intracerebral or intraocular lymphomas [2]. Early, fast and precise diagnosis of CNS DLBCL is a prerequisite for prompt and proper treatment and improved patient outcomes [1]. Unfortunately, persistent and unexplained neurologic symptoms, imaging features, and sensitivity to glucocorticoids of the intracranial lesions are shared between different pathologies, including CNS DLBCL and various non-malignant CNS lesions (n-ML), neurological disorders among them [3,4]. For establishing diagnosis of CNS DLBCL, stereotactic biopsy is a gold standard. There is a number of additional diagnostic possibilities, including imaging and cytological and flow cytometry (FCM) examination of cerebrospinal fluid (CSF) [4]. FCM has some diagnostic limitations due to the CNS DLBCL biology, with CSFs commonly lacking the malignant cells in the CSF. New CSF markers for CNS DLBCL, including microRNAs -21, -19b, and -92a, RNU2-1f, CXCL13, interleukins −6, −8, and −10, soluble interleukin-2-receptor, soluble CD19, soluble CD27, tumor necrosis factor-alfa, beta-2-microglobulin, antithrombin III, soluble transmembrane activator and calcium modulator and cyclophilin ligand interactor, soluble B cell maturation antigen, neopterin and osteopontin and three markers in blood [5,6,7,8], CXCL-13, beta-2-microglobulin and neopterin were found to present the highest potential in diagnosing CNS lymphoma [8], but their utility for accurate differential diagnosis has not been finally established. Thus, the accurate differential diagnosis of CNS DLBCL remains a significant challenge.

Aberrant expression of microRNAs, small, non-coding RNA molecules that regulate gene expression, contribute to various pathologies. Thus, microRNAs have emerged as promising biomarkers, also in lymphoid malignancies and neurologic diseases [9,10]. microRNAs display conserved tissue-specific distribution, and miR-9, miR-125b, and let-7b are known to be brain-enriched [11,12,13,14]. CSF and circulating miR-15b has been proposed as glioma biomarkers, while miR-29a and miR-29b as Alzheimer’s disease biomarkers [15,16,17]. Deregulated miR-20a-5p expression has been linked to the pathogenesis of multiple sclerosis (MS) [18]. Increased expression of miR-196b and miR-155 play a significant role in some types of leukemia and in diffuse large B cell lymphoma, not otherwise specified (DLBCL, NOS), respectively [19,20,21,22,23]. A diagnostic value of CSF miR-21, miR-19b, and miR-92a assessment for the differential diagnosis of PCSNL and neurological disorders, has been suggested [24]. So far, concurrent expression of microRNAs in diagnostic brain biopsy and the relevant CSF samples from patients with PCNSL and with n-ML has not been studied.

We measured the expression of miR-9, miR-19b, miR-21, miR-92a, miR-125b, miR-155, miR-196b, and let-7b in the archival formalin-fixed paraffin-embedded tissue (FFPET) from brain tumors and in CSF samples, in order to evaluate the potential of the CSF and tumor miRNAs as biomarkers to assist the differential diagnosis of CNS DLBCL vs. n-ML.

## 2. Results

### 2.1. miR-155, miR-196b, miR-9, miR-125b, and miR-Let-7b Levels in FFPET Brain Biopsies and CSF Samples from Patients with CNS, DLBCL vs. n-ML

**Figure 1:**
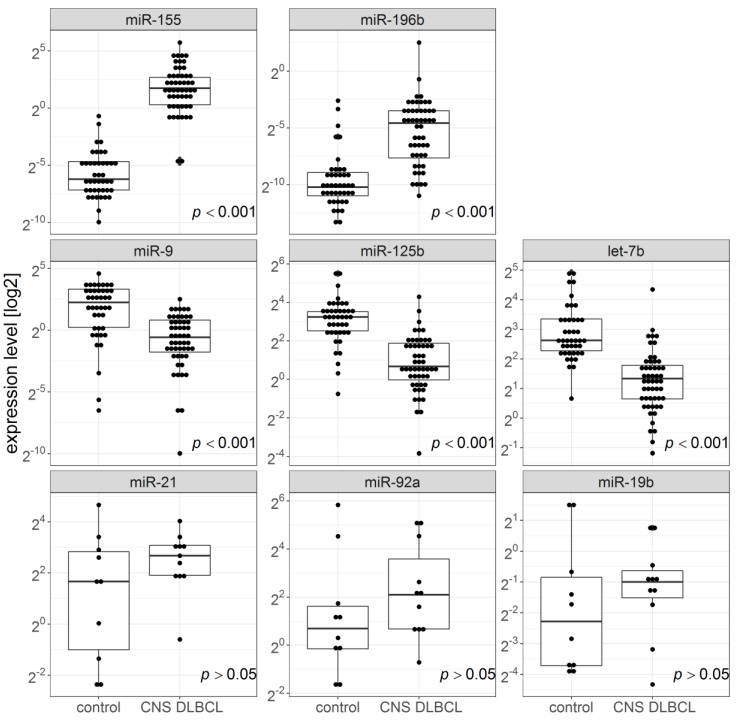
MicroRNA expression in formalin-fixed paraffin-embedded tissue samples of cerebral lesions from patients with CNS DLBCL (*n* = 52 for the top and middle rows, and *n* = 11 for the bottom row) and with non-malignant brain lesions (*n* = 42 for the top and middle rows, and *n* = 10 for the bottom row).

Brain FFPET samples (Figure 1) showed significantly higher miR-155 and miR-196b expression in CNS DLBCL than in n-ML (median miR-155 expression: 3.353 and 0.0135, respectively, *p* = 6.03 × 10^−16^; median miR-196b expression: 0.041 and 0.00085, respectively, *p* = 1.27 × 10^−9^). The expression of miR-9, miR-125b, and miR-let-7b was significantly lower in FFPET samples of CNS DLBCL as compared to that in non-malignant CNS lesions (median miR-9 expression: 0.67 and 4.77, respectively; *p* = 1.7 × 10^−7^; median miR-125b expression: 1.59 and 9,46, respectively; *p* = 1.01 × 10^−10^; median miR-let-7b expression: 2.53 and 6.18, respectively; *p* = 5.4 × 10^−11^).

Contrary to FFPET miR expression, there were no differences in the CSF levels of miR-9, miR-9*, miR-125b, miR-155, and miR-196b between patients with CNS DLBCL and with n-ML (median expressions in CNS DLBCL and n-ML: miR-9, 0.0555 and 0.0561, respectively, *p* = 0.931; miR-9*, 0.780 and 0.407, respectively, *p* = 0.493; miR-125b, 4.460 and 3.790, respectively, *p* = 0.771; miR-155, 0.020 and 0.053, respectively, *p* = 0.201; miR-196b, 0.020 and 0.040, respectively; *p* = 0.483).

**Figure 2:**
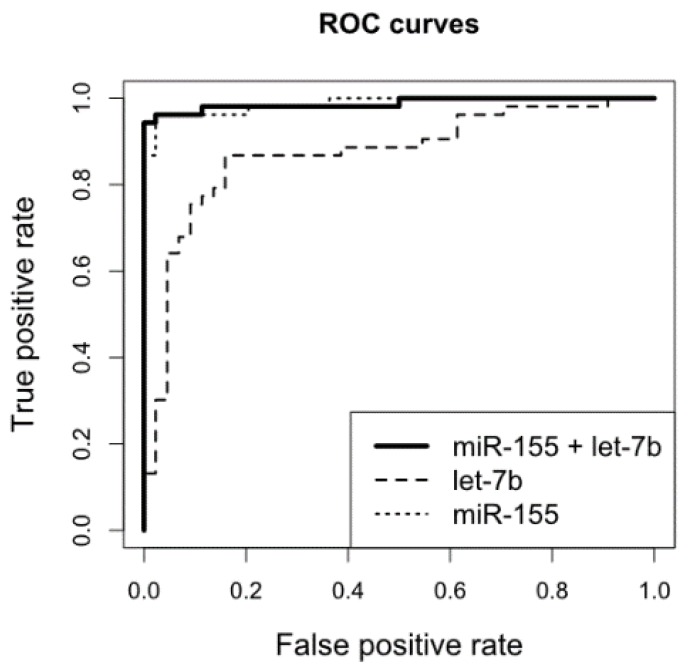
ROC analysis of the performance of a combined miR-155 and miR-let-7b expression in brain biopsy samples to discriminate CNS DLBCL and non-malignant CNS lesions.

As shown by the ROC analysis (Figure 2), combined miR-155 and miR-let-7b expression levels in brain FFPET samples presented the best discrimination power between CNS DLBCL and n-ML (98% specificity and 96% sensitivity, AUC = 0.988). Supplementing the combination of miR-155 and miR-let-7b with miR-196b or miR-125b did not improve the discrimination power. The following logistic regression model was built based on a combination of miR-let-7b and miR-155 expression levels to predict CNS DLBCL diagnosis: alpha = −2.664 − 0.1225[miR-let-7b] + 9.32[miR-155].

To validate the model, i.e., the discrimination power of miR-155 and miR-let-7b expression measurements, an independent, blinded set of brain biopsies was examined. Out of 17 samples of CNS DLBCL, and 17 samples of n-ML, all but one were correctly classified (Table S1). The falsely classified one was a CNS DLBCL sample predicted as a n-ML. However, further detailed analysis of this FFPET sample revealed it contained almost exclusively normal cells of adjacent tissue, thus the sample was not representative for the tumor. After the validation step that showed highly consistent results between the study and validation groups (Figures S1 and S2), data were pooled according to the final diagnosis (CNS DLBCL vs. n-ML) and the pooled series were statistically re-analyzed. Figure 1 shows the results of the pooled data.

### 2.2. miR-21, miR-19b, and miR-92a Levels in CSFs and Brain Biopsies from Patients with CNS DLBCL vs. n-ML

**Figure 3:**
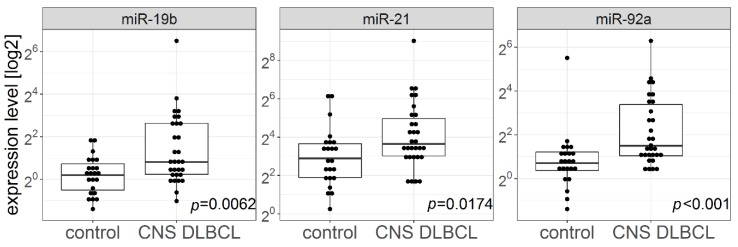
MicroRNA levels in cerebrospinal fluids from patients with non-malignant cerebral lesions (controls) (*n* = 23) and with CNS DLBCL (*n* = 30).

The levels of CSF miR-21, miR-19b, and miR-92a (Figure 3) were significantly higher in patients with CNS DLBCL than in patients with n-ML (miR-21: median 12.628 and 6.804, respectively, *p* = 0.016738; miR-19b: median 1.759 and 1.139, respectively, *p* = 0.006055; and miR-92a: median 2.820 and 1.628, respectively, *p* = 0.001135).

**Figure 4:**
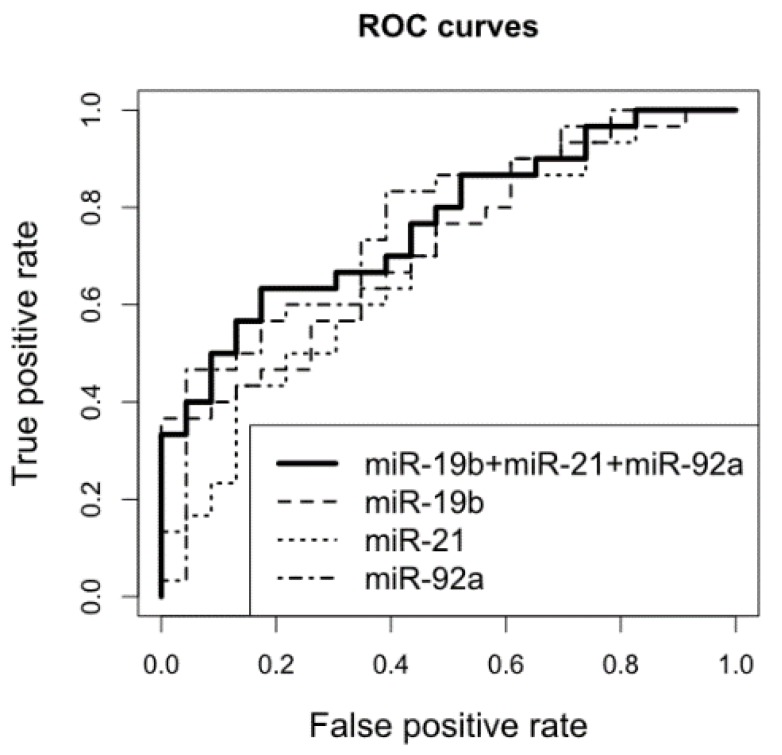
Performance of CSF miR-19b, miR-21, and miR-92a expression alone and in combination, to discriminate patients with non-malignant cerebral lesions (controls, *n* = 23) and with CNS DLBCL (*n* = 30).

Next, based on a combination of CSF miR-19b, miR-21, and miR-92a levels, we built the the following logistic regression model: alpha = −1.338 + 0.707[miR-19b] + 0.0261[miR-21] − 0.0857[miR-92a]. We found that a combination of CSF miR-21, miR-19b, and miR-92a measurements (AUC 0.714, 0.737, and 0.771, respectively) discriminated patients with CNS DLBCL from patients with n-ML with a specificity 80.77% and a sensitivity of 63.33% (Figure 4).

miR-21, miR-19b, and miR-92a expression was also examined in 11 FFPET samples of CNS DLBCL and 10 of n-MLs, including the matched CSF–FFPET samples, obtained from 6 CNS DLBCL patients. Contrary to CSF samples, miR-21, miR-19b, and miR-92a expression in the FFPET samples did not significantly differ between CNS DLBCLs and n-MLs (Figure 1) (median miR-21 expression: 6.4 and 3.19, respectively; *p* = 0.15; median miR-19b expression: 0.5 and 0.22, respectively; *p* = 0.39; median miR-92a expression: 4.28 and 1.67, respectively; *p* = 0.17).

The following miRs, miR-9*, miR-15b, miR-20b, miR-29a, miR-29b-1, and miR-29b-2 were found to be expressed at similar levels in FFPET samples of CNS DLBCL (*n* = 11) and of n-ML (*n* = 10).

### 2.3. Survival Analyses

The overall survival of patients with CNS DLBCL is shown in Figure S3a. There were no significant differences in OS related to tumor microRNA expression levels (Figure S3b).

### 2.4. CNS DLBCL Immunophenotypes and miR Expression

We found no miR expression to be related to the immunophenotype subtypes of the CNS DLBCL.

## 3. Discussion

We showed that FFPET samples presented significantly overexpressed miR-155 and miR-196b, while miR-9, miR-125b, and miR-let-7b were downregulated in CNS DLBCL, as compared to n-ML. We further validated these results in an independent set of samples and demonstrated that a combined assessment of miR-155 and miR-let-7b discriminated CNS DLBCL from n-ML with 97% accuracy. We are the first to show that microRNA expression in brain biopsy samples differentiates CNS DLBCL from n-ML. On top of that, a significance of cell content assessment in the samples was brought up. This could be useful for clinical practice when routine histopathology and immunochemistry is misleading [25,26], and in the biopsies of the so-called vanishing tumors, observed following steroid treatment (based on our own experience).

The increased expression of brain-specific miR-9 and miR-125b in n-ML may relate to their crucial role in a number of neurogenic processes, including microglial migration, regulation of the adult neural stem cells quiescence and activation, axonal branching and outgrowth, and astrogliosis during neurodegeneration [27,28,29,30]. The potent neurogenic role of miR-9 has been confirmed by the ectopic expression of miR-9/miR-124 in human adult fibroblasts. It triggered chromatin accessibility reconfiguration, DNA methylation, and mRNA expression changes, and induced a default neuronal state, and the conversion of fibroblasts to functional neurons [31]. Moreover, deregulated miR-9, miR-125b, and let-7b expression has been implicated in the pathogenesis of many neurologic diseases, including schizophrenia, Alzheimer disease, amyotrophic lateral sclerosis, Huntington’s disease, ischemic stroke, and MS [32,33,34,35,36,37,38,39,40,41], what further pointed to the relevance of those miRNAs in brain tissues.

The increased expression of two inflammation- and lymphoma-associated miRNAs, miR-155 and miR-196b, in CNS DLBCL compared to n-ML, is in line with previously published data. PCNSL has been shown to express higher levels of miR-155 in than nodal DLBCL, NOS specimens [42,43]. miR-196b has been demonstrated to contribute to the pathogenesis of some types of leukemia [22], and, in acute myeloid leukemia, high miR-196b expression influenced prognosis [44].

Immunophenotypic subgroups of CNS DLBCL (CD5-positive, germinal-center B-cell (GCB)-type and non-germinal center B-cell (non-GCB)-type) presented no differences in the examined miRNA profiles of both CSF and FFPET specimens. Similarly, none of the microRNAs examined in the tumors related to OS in patients with CNS DLBCL. Takashima et al. [45] have recently suggested a miRNA expression signature of PCNSL tumors as a predictor of prognosis. However, the signature comprised other microRNAs that we examined here.

CSF microRNAs have been considered as novel potential biomarkers for patients with brain lesions [10]; still, the available data are scarce and further validation is necessary. While examining the CSFs, we found a significantly higher levels of miR-21, miR-19b, and miR-92a in patients with CNS DLBCL than in patients with n-ML, and no differences between the two series of patients in the CSF levels of miR-9, miR-125b, miR-155, and miR-196b. A set of CSF miR-21, miR-19b, and miR-92a differentiated CNS DLBCL from n-ML, with a specificity of 80.77% and a sensitivity of 63.33%. In PCNSL, Baraniskin et al. [24,46] were the first to demonstrate CSF miRNA assessment as a powerful tool for the diagnosis and follow-up of patients. In line with our results, they showed the increased levels of miR-21, miR-19b, and miR-92a [24]. However, the diagnostic accuracy they presented was much higher (95.7% sensitivity and 96.7% specificity). The reason of this discrepancy most probably lies in the reference groups. The control series we used comprised patients with benign brain neoplasms and diverse neurological disorders, while their series were dominated by MS cases. Notably, patients with MS have recently been shown to present diminished CSF miR-21 levels [47]. Considering the above, the diagnostic performance achieved by Baraniskin et al. [24] for CSF levels of miR-21, miR-19b, and miR-92a may have been exceedingly high. Consistently with our results on miR-125b, Drusco et al. [48] found no differences in the levels of CSF miR-125b between patients with PCNSL and n-ML, while there was a significant up-regulation of miR-125b in medulloblastoma and glioblastoma. It should be noted that although Drusco et al. [48] employed high throughput methods, in their series of patients with benign and malignant brain tumors of different origin, only 3 were diagnosed with PCNSL. CSF miR-155 levels previously studied in glioma patients were found not to differ from that of non-malignant controls, although a TCGA data analysis showed over 2-fold tumor tissue miR-155 overexpression [49].

Interestingly, the examined miRNA levels in CSFs and tissues from patients with CNS DLBCL did not match. Similar CSF patterns of miR-155, miR-196b, miR-9, and miR-125b, and different patterns of miR-21, miR-19b, and miR-92a between patients with n-ML and those with CNS DLBCL did not reflect the miR patterns in brain tumor samples. It is not clear why in the CNS DLBCL, CSFs, and the relevant biopsy samples are characterized by different microRNA profiles. We believe that inconsistencies between tumor and CSF miRNA expression were not linked to RNA degradation characterizing FFPET samples. A number of studies have proven FFPET-derived miRs to have enhanced stability [50]. Moreover, the inconsistencies concerned were also less prone to degradation, GC-rich miR-92a [51]. To explain the discrepancies between miR profiles of CSFs and tumors, one may speculate that while microRNAs detected in the brain biopsy specimens may originate mainly from lymphoma cells, CSF microRNAs may derive from other cell types besides lymphoma cells, such as those associated with the ventricular choroid plexus, ventricular system, the subarachnoid space, and spinal cord [52]. Moreover, since CNS DLBCL and neurodegenerative diseases are frequently accompanied by a blood–brain barrier dysfunction [53,54], circulating microRNAs may also contribute to disease-related CSF microRNA profiles. Interestingly, as recently reported, in healthy donors, the miRNA profiles of brain tissues and CSF exosomes were not identical, suggesting a selective secretion of miRNAs by brain tissues [55]. For example, brain-enriched miR-124a that has been identified in CSFs of patients with glioblastoma and CNS metastases, was not expressed in the tumor samples [49]. Also, proteome studies have shown discrepancies between CSF and the relevant PCNSL tissue expression [53]. Inconsistencies in microRNA profiles have also been observed between peripheral tumor specimens and the respective blood samples [56]. Interestingly, an inverse correlation between circulating and tumor-associated microRNA levels has also been observed [57]. Therefore, in line with previous suggestions by Witwer [58] that referred to circulating microRNAs in solid tumor patients, one cannot exclude that CNS DLBCL- or non-malignant disease-related changes in CSF microRNA levels may reflect a systemic response to the pathologies rather than deregulations in the brain lesion.

Overall, we showed here that the expression profile of tissue miR-155, miR-196b, miR-9, miR-125b, and miR-let-7b differentiated CNS DLBCL from n-ML. We proposed and validated the following logistic regression model to discriminate between PCNSL and non-malignant brain lesions: alpha = −2.664 − 0.1225[miR-let-7b] + 9.32[miR-155], with an index of >0 for samples predicted as malignant. We also confirmed the utility of miR-21, miR-19b, and miR-92a as CSF CNS DLBCL markers, but of lower diagnostic accuracy than previously reported (see Table S2) [24].

To further investigate microRNA profiles of biopsy tissues and CSFs of patients with CNS lesions, in order to potentially identify new microRNA molecules involved, to look into the biological contexts, and to verify the utility microRNA assessment of as an ancillary tool in the differential diagnosis and follow up of CNS DLBCL patients, we are currently using next generation sequencing approaches.

## 4. Materials and Methods

### 4.1. Patients and Samples

#### 4.1.1. Patients

Brain FFPET (Tables S3 and S4) and CSF (Tables S2 and S5) samples of CNS DLBCL and n-ML were collected for routine diagnostic purposes from 131 patients diagnosed and consulted at the Department of Pathology and Laboratory Diagnostics. All samples were collected at the initial diagnosis. CNS DLBCL patients were treated at the Department of Lymphoid Malignancies, Maria Sklodowska-Curie Institute—Oncology Center in Warsaw (Tables S3 and S5, patients no. 1–34) and in other Warsaw hospitals (Tables S3 and S5, patients no. 35–52), between 2010 and 2017. CSF samples of patients with n-ML (Table S2) were collected in Warsaw neurological hospitals for routine FCM diagnosis performed at the Flow Cytometry Laboratory, Department of Pathology and Laboratory Diagnostics, Maria Sklodowska-Curie Institute—Oncology Center in Warsaw between 2013 and 2016.

#### 4.1.2. Sample Collection

CSF samples were obtained via lumbar puncture from patients with the initial clinical and/or MRI presentation suggesting PCNSL, and subsequently diagnosed with CNS DLBCL (*n* = 30, 15 women/15 men, median age 59, range 20–79, Table S5) or with a final diagnosis of n-ML (*n* = 23, 13 women/10 men, median age 31, range 9–81, Table S2). The CSF samples were centrifuged at 170× *g*, to recover the cells for the routine cytological and FCM examinations. The leftover supernatants were centrifuged at 500× *g* for 10 min at 20 °C, aliquoted to 400 µL volumes, and stored at −70 °C.

Brain tumor biopsy samples were obtained by stereotactic biopsy or surgical resection of CNS tumors of patients with the initial clinical and/or MRI presentation suggesting PCNSL, and subsequently diagnosed with CNS DLBCL (*n* = 52, 32 women/20 men, median age 62, range 31–82, Table S3) or with n-ML (*n* = 42, 18 women/24 men, median age 47, range 18–78, Table S4). The first series of consecutive tumor samples from patients with CNS DLBCL (*n* = 35) and n-ML (*n* = 23) served as study groups, subsequent samples were blinded and served as a validation group for miRNA evaluation. The validation group included 17 CNS DLBCL, and 17 n-ML. Additionally, the study and validation series were pooled and re-analyzed.

#### 4.1.3. Diagnostic Procedures: Immunohistochemical Staining of FFPET Samples

FFPET samples were prepared by routine methods. For histopathological evaluation, hematoxylin and eosin staining was performed. For immunohistochemistry (IHC) tissue sections were incubated with the diluted antibodies for 1 h in Omnis autostainer (Dako, Glostrup, Denmark) following antigen-retrieval technique, if necessary, using the EnVision™ Detection Systems FLEX kit (Dako Corp, Carpinteria, CA, USA, code K 8000) and monoclonal antibodies (MoAbs) specific for: CD20, CD10, BCL6, MUM1, CD5, BCL2, Ki-67 (Table S6), as previously described [23]. For the Ki-67 index assessment, 200 cells were counted under HPF (×400) examination, in each case. The proliferative fraction, as detected by Ki67 staining, was high (usually over 90%).

For more details see Supplementary Methods.

#### 4.1.4. Diagnostic Procedures: Immunophenotyping, Morphology and Proliferation Evaluation of CSF Cells

Immunophenotyping of CSF samples was performed by FCM. Concentrated cells isolated from CSFs by centrifugation were incubated with a panel of MoAbs (for staining procedure see [23], for a list of MoAbs see Table S7). For more details see Supplementary Methods.

Simultaneously, CYT were stained with a May–Grünwald–Giemsa for morphological evaluation.

#### 4.1.5. Final Diagnosis

To set the final diagnosis considered in this study, all malignant and non-malignant HP/IHC biopsy specimens and FCM analyses were re-evaluated in 2018 by an experienced pathologist (GR) in the context of clinical characteristics, imaging results, and individual patient histories followed for several years. The final diagnosis of CNS DLBCL and n-ML considered histopatological (HP) criteria and IHC examination according to the 2017 WHO classification [2], and included immunohistochemical subgroups, CD5-positive, GCB- and non-GCB-types, distinguished by the Hans algorithm, i.e., based on CD10, BCL6, and multiple myeloma oncogene-1 (MUM1) expression, as proposed in the previous WHO 2008 classification [25]. For more details see Supplementary Methods.

In each case, systemic DLBCL involvement was ruled out. Only non-immunocompromised patients were included in this study. The clinical and pathomorphological characteristics of the patients are presented in Tables S2–S5.

The study was approved by the local Institutional Review Board (decision no. 4/2011/1/2012).

### 4.2. RNA Extraction and RT-qPCR

Total RNA was isolated from CSF samples with the Gene Matrix Universal RNA/miRNA Purification Kit (EURx, Gdansk, Poland), according to the manufacturer’s instructions, with some minor modifications specified herein. Three hundred µL of the RL buffer (EURx) was added directly to the 400 µL of a frozen CSF sample. After complete thawing, the sample was vortexed, and 500 µL of the acid phenol:chloroform:IAA (Ambion, Foster City, CA, USA) was added. The sample was mixed briefly and centrifuged at 11.000× *g* for 3 min. Subsequently the upper aqueous phase was transferred to a homogenization column and centrifuged (11.000× *g*, 2 min). In addition, after spinning, following a suggestion of Alexander Baraniskin (personal communication), first, 60 µg of glycogen (Invitrogen, Carlsbad, CA, USA) was added to the flow-through; secondly, the Reverse Transcription (RT) reaction was performed using 10 µL, as the amounts of the isolated total RNA were below the sensitivity of the NanoDrop ND 1000 Spectrophotometer (NanoDrop Technologies, ThermoFisher Scientific, Madison, WI, USA).

Ten 20-µm thick sections of each FFPET sample were cut with a disposable blade. Total RNA was extracted using the RecoverAll™ Total Nucleic Acid Isolation Kit (Applied Biosystems, Foster City, CA, USA), according to the manufacturer’s recommendations. RNA concentration and purity were measured with the NanoDrop ND 1000 Spectrophotometer (NanoDrop Technologies).

The RT reaction was performed for all samples using the TaqMan MicroRNA Reverse Transcription Kit (Applied Biosystems), using 10 µL of total RNA, as specified by Baraniskin [24]. For the microRNA analysis, the following specific TaqMan MicroRNA Assays (Applied Biosystems cat. no. 4427975) were used: miR-9-5p (ID 000583), miR-9-3p* (ID 002231), miR-let-7b-5p (ID 000378), miR-15b-5p (ID 000390), miR-19b-3p (ID 000396), miR-20b-5p (ID 001014), miR-21-5p (ID 000397), miR-29a-5p (ID 002447), miR-29b-1-5p (ID 002165) and miR-29b-2-5p (ID 002166), miR-92a-3p (ID 000431), miR-125b-5p (ID 000449), miR-155-5p (ID 000479), miR-196b-5p (ID 002215), and as the internal control, miR-24-3p (ID 000402). MicroRNA sequences with GC content are given in Table S8. Quantitative real-time polymerase chain reaction (qPCR) was performed on a 7500 Fast Real-Time PCR System (Applied Biosystems). All PCR reactions were carried out in triplicates at a final volume of 10 µL. The data were analyzed with the 7500 Software v.2.0.6 (Applied Biosystems) and the relative miRNAs quantities were calculated with the 2^−ΔCt^ method. Due to limitations in sample amounts, not all miRs were assessed in all samples.

### 4.3. Statistical Analyses

The differences in miRNA expression levels between samples (both in CSF and FFPET) were assessed by the Mann–Whitney *U* test. Associations of miRNA expression and clinical variables were probed with the Kruskal–Wallis test.

The Cox proportional hazards model was used to analyze the correlation of clinical data and microRNA levels with overall survival (OS). A separate model was created for each miR variable. A multivariate model was applied to correlate OS with all three treatments. OS was calculated from the date of diagnosis to the date of death, or—for patients who were still alive—to the date of the last observation. A logistic regression model was built to predict diagnosis on the basis of miR-let-7b and miR-155 expression levels in FFPET samples of the study set and on the basis of miR-19b, miR-21, and miR-92a levels in the whole set of CSF samples.

The analyses were performed in R (version 3.4.1) with the aid of the survival package (version 2.42-6).

## 5. Conclusions

- Tumor miR-155, miR-196b, miR-9, miR-125b, and miR-let-7b expression levels are significantly different in PCNSL and in non-malignant brain lesions.
- A logistic regression model is proposed to discriminate between PCNSL and non-malignant brain lesions.
- We confirm the value of cerebrospinal fluid miR-21, miR-19b, and miR-92a profiles as potential CNS DLBCL markers.
- PCNSL CSFs and the relevant biopsy samples are characterized by specific, different microRNA profiles.
- The examined microRNA profiles do not influence overall survival of PCNSL patients.

## Supplementary Materials

The following are available online at https://www.mdpi.com/2072-6694/11/11/1647/s1, Figure S1: MicroRNA expression in formalin-fixed paraffin-embedded brain tumor biopsy samples of patients with CNS DLBCL and with non-malignant CNS diseases (Controls)., Figure S2: MicroRNA expression in formalin-fixed paraffin-embedded brain tumor biopsy samples of patients with CNS DLBCL and with non-malignant CNS diseases., Figure S3: Overall survival (OS) analyses of patients with CNS DLBCL., Table S1: Validation of the discrimination power of miR-155 and miR-let-7b expression measurements between CNS DLBCL and non-malignant tumors. Table S2: Patients with non-neoplastic cerebral tumors (*n* = 23): clinical diagnosis and miRNA expression level in the cerebrospinal fluid (CSF)., Table S3: Clinical and pathomorphological characteristics of CNS DLBCL patients (*n* = 52), along with miRNA expression levels in tumor biopsy samples. Table S4: Non-neoplastic cerebral or cerebellum tumor patients (*n* = 42): pathomorphological diagnosis and tumor miRNA expression levels. Table S5: Patients with CNS DLBCL (*n* = 30): clinical diagnosis and miRNA expression level in the cerebrospinal fluid (CSF). Table S6: The clones of Dako antibodies used for immunohistochemistry analyses. Table S7: Antibodies used for flow cytometry. Table S8: MicroRNA sequences with GC content.
