# Supplementary material for: Tumor and Cerebrospinal Fluid microRNAs in Primary Central Nervous System Lymphomas
Source: Cancers (Basel). 2019 Oct 25;11(11):1647. doi: 10.3390/cancers11111647 (PMC6895823; doi:10.3390/cancers11111647)
Supplement: Supplementary file 1 [file cancers-11-01647-s001.zip › Suppl ZIP_PROOFS/Figure S1.pdf]

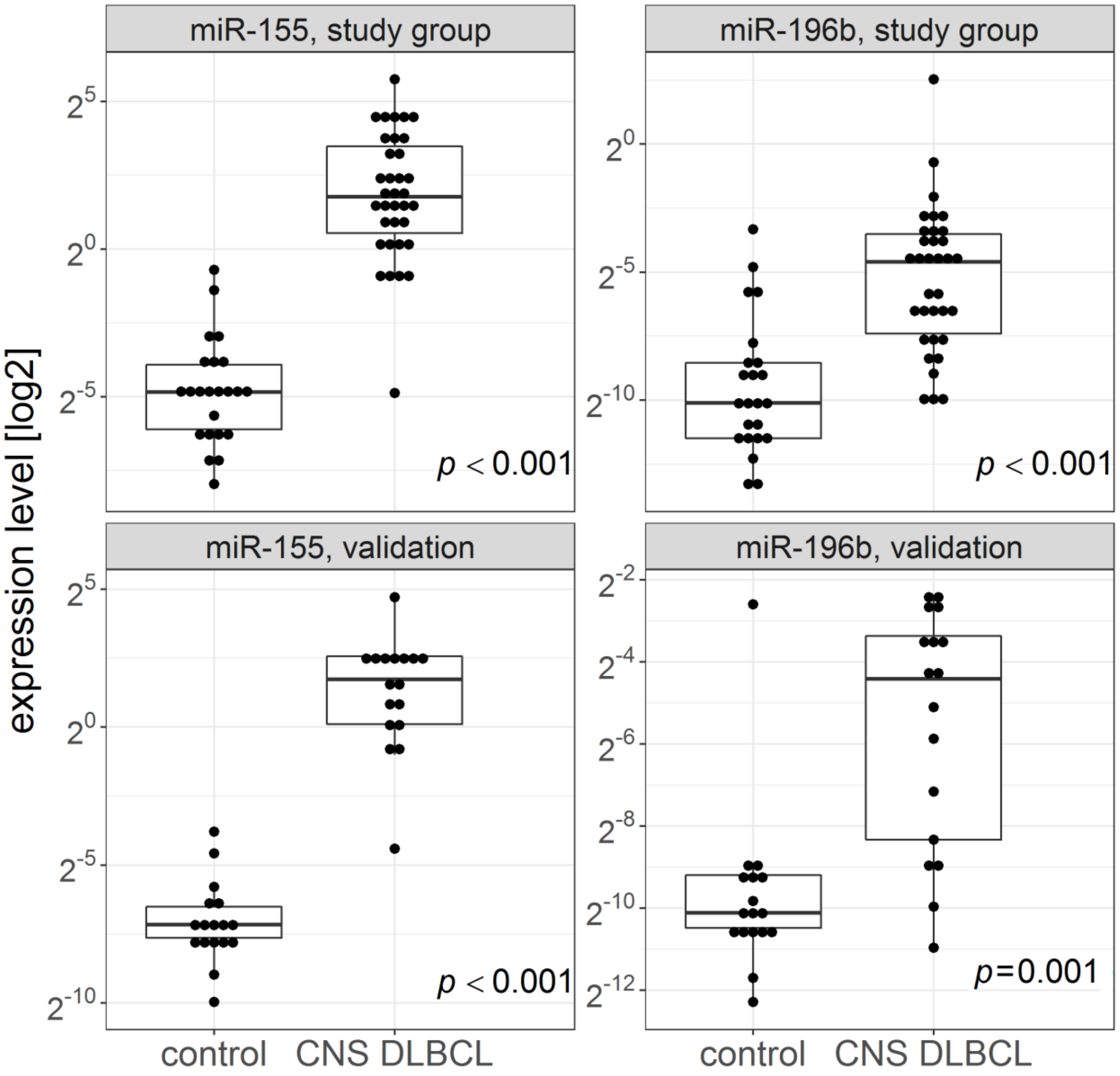

Figure S1. MicroRNA expression in formalin-fixed paraffin-embedded brain tumor biopsy samples of patients with CNS DLBCL and with non-malignant CNS diseases (Controls). Top row, study groups, n=58, including CNS DLBCL, n=35 and controls, n=23. Median expression values in control groups and CNS DLBCLs: for miR-155 0.035 and 3.395, for miR-196b, 0.0009 and 0.04 respectively. Bottom row, validation sets, n=34, including CNS DLBCL, n=17, and controls, n=17. Median expression values in control groups and CNS DLBCLs: for miR-155 0.007 and 3.31, respectively for miR-196b, 0.0009 and 0.047, respectively.
