# Supplementary material for: Tumor and Cerebrospinal Fluid microRNAs in Primary Central Nervous System Lymphomas
Source: Cancers (Basel). 2019 Oct 25;11(11):1647. doi: 10.3390/cancers11111647 (PMC6895823; doi:10.3390/cancers11111647)
Supplement: Supplementary file 1 [file cancers-11-01647-s001.zip › Suppl ZIP_PROOFS/Figure S2.pdf]

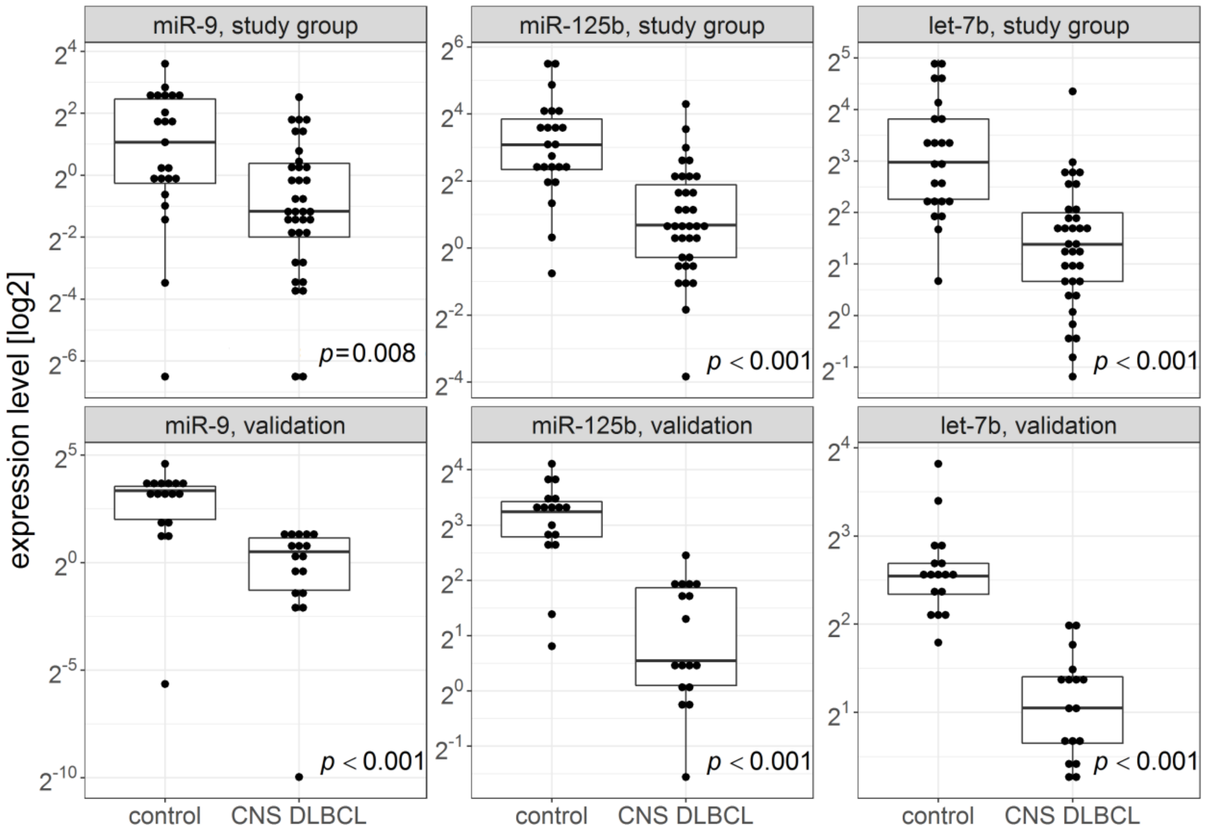

Figure S2. MicroRNA expression in formalin-fixed paraffin-embedded brain tumor biopsy samples of patients with CNS DLBCL and with non-malignant CNS diseases (control). Top row, study groups: CNS DLBCL, n=35 and controls, n=23; median expression values in control groups and CNS DLBCLs for miR-9, 2.097 and 0.446, respectively, for miR-125b, 8.47 and 1.61 for miR-let-7b, 7.873 and 2.61, respectively. Bottom row, validation group, CNS DLBCL, n=17, controls, n=17; median expression values in control group and CNS DLBCLs for miR-9, 10.08 and 1.42, respectively, for miR-125b, 9.47 and 1.46, respectively and for miR-let-7b, 5.84 and 2.07, respectively.
