# Supplementary material for: Tumor and Cerebrospinal Fluid microRNAs in Primary Central Nervous System Lymphomas
Source: Cancers (Basel). 2019 Oct 25;11(11):1647. doi: 10.3390/cancers11111647 (PMC6895823; doi:10.3390/cancers11111647)
Supplement: Supplementary file 1 [file cancers-11-01647-s001.zip › Suppl ZIP_PROOFS/Figure_S3.pdf]

a.

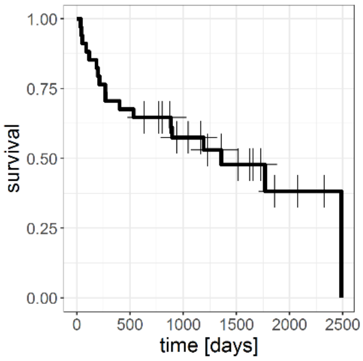

b.

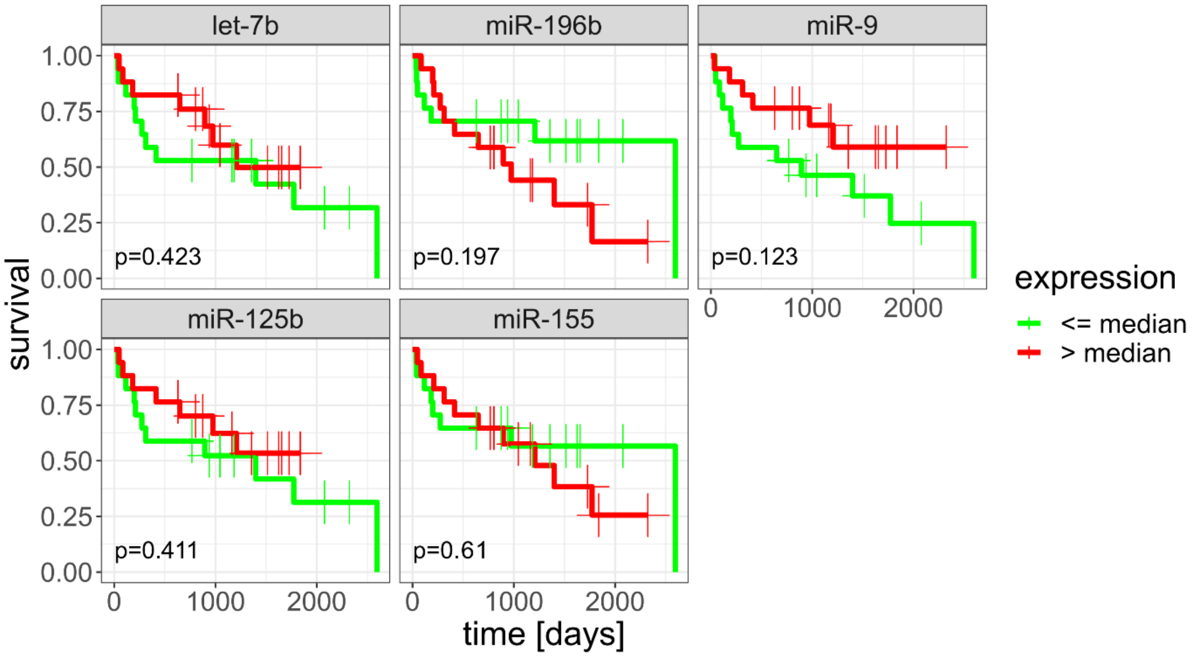

Figure S3. Overall survival (OS) analyses of patients with CNS DLBCL (patients no 1-34 in Table S3). Cox proportional hazards model was applied. A separate model was created for each variable OS was calculated from the date of diagnosis to the date of death, or – for patients who were still alive – to the date of the last observation. **a**. OS of all 34 patients; **b**. OS depending on tumor microRNA expression levels.
