# Supplementary material for: Tumor and Cerebrospinal Fluid microRNAs in Primary Central Nervous System Lymphomas
Source: Cancers (Basel). 2019 Oct 25;11(11):1647. doi: 10.3390/cancers11111647 (PMC6895823; doi:10.3390/cancers11111647)
Supplement: Supplementary file 1 [file cancers-11-01647-s001.zip › Suppl ZIP_PROOFS/Supplementary Methods.pdf]

Zajdel. et al., Cancers 2019, Supplementary Methods

Immunohistochemical staining of FFPET samples

A reaction for CD20, BCL6, MUM1 and CD5 was considered positive if at least 20% of the CNS DLBCL cells showed staining, while for CD10 – if any cell showed staining. Cases with a 100% IHC positivity of tumor cells were evaluated as positive (+). Patterns of BCL2 staining were divided into 3 categories: (–) "negative", the lack of BCL2 expression on CNS DLBCL cells, (+) "positive", the expression of BCL2 on CNS DLBCL cells, comparable or lower than in the surrounding small lymphocytes, and (++)h, strong BCL2 staining on CNS DLBCL cells, higher than in the background cells. A reaction was considered positive, if at least 20% of the CNS DLBCL cells showed BCL2 signal.

Flow cytometry immunophenotyping of CSF samples

The expression of B-cell antigens CD(45/19/20/10) with kappa/lambda light chain, T-cell antigens CD(45/3/4/8/5/43) and macrophage antigens (CD14, HLADR) was quantified on FACSCalibur and FACSCanto II cytometers (Becton Dickinson, BD) and samples were categorized into three groups, according to the percentages of positive cells: (–), no expression (<20% of neoplastic cells); (+/–), expression in ≥20%<100% of cells; (+), expression in 100% of cells.

Immunohistochemical subgroups of CNS DLBCL

In 52 CD20(+) CNS DLBCL cases, 15% (8/52) expressed CD10, 94% (49/52) – BCL6, 100% (52/52) – MUM1, 26% (12/47) – CD5 and 98% (51/52) – BCL2. BCL2 was overexpressed (BCL2higher) in 37% of cases. CNS DLBCLs have been classified as non-GCB (35 cases, 67%), GCB (5 cases, 10%) and CD5 positive (12 cases, 23%).
