# Supplementary material for: Tumor and Cerebrospinal Fluid microRNAs in Primary Central Nervous System Lymphomas
Source: Cancers (Basel). 2019 Oct 25;11(11):1647. doi: 10.3390/cancers11111647 (PMC6895823; doi:10.3390/cancers11111647)
Supplement: Supplementary file 1 [file cancers-11-01647-s001.zip › Suppl ZIP_PROOFS/Table_S_1.pdf]

Table S1. Validation of the discrimination power of miR-155 and miR-let-7b expression measurements between CNS DLBCL and non-malignant tumors. Following the study group analysis (**a**, n=58), an independent series of biopsies of CNS DLBCL and non-malignant tumors was examined (validation group, **b**, n=34).
To predict diagnosis on the basis of miR-let-7b and miR-155 levels, a logistic regression model was built: alpha= –2.664–0.1225[miR-let-7b] + 9.32[miR-155]. Samples with a prediction index of >0 were predicted malignant. The incorrectly qualified sample is marked in yellow.
(**a**)

patient ID no.*	true malignant (1) vs. non-malignant (0)	miR-let-7b	miR-155	prediction index
14	1	1.98	53.291	493.7694
16	1	3.57	25.7	236.4242
38	1	1.57	23.72	218.2155
15	1	3.2	22.246	204.278
11	1	6.52	19.136	174.8858
39	1	7.88	18.993	173.3863
9	1	4.17	15.103	137.5858
36	1	2.61	12.79	116.2196
19	1	0.89	11.874	107.8932
10	1	2.3	10.406	94.03853
44	1	3.09	8.365	74.91944
37	1	2.53	5.938	52.36822
4	1	2.63	5.38	47.15537
21	1	3.83	5.224	45.5544
43	1	0.75	4.661	40.68459
18	1	1.22	3.997	34.43846
2	1	0.72	3.48	29.68125
22	1	3.19	3.395	28.58639
17	1	0.57	3.11	26.2512
8	1	1.4	3.007	25.18953
20	1	1.59	2.619	21.55005
40	1	2.22	2.512	20.47561
35	1	1.92	2.43	19.74812
3	1	5.54	2.05	15.76293
12	1	0.44	1.943	15.39059
1	1	1.94	1.71	13.03522
6	1	1.05	1.243	8.79179
24	1	7.05	1.168	7.3576
5	1	4.18	1.077	6.861136
7	1	3.13	1.007	6.337387
13	1	1.59	0.51	1.894004
42	1	3.37	0.529	1.852981
23	1	6.23	0.534	1.549144
73	0	3.874	0.382	0.421174
41	1	20.45	0.555	0.002481
75	0	27.868	0.615	-0.34725
64	0	1.588	0.132	-1.62874
71	0	3.724	0.125	-1.95571
63	0	4.459	0.064	-2.61429
69	0	5.621	0.078	-2.62619
70	0	3.185	0.041	-2.67255
57	0	4.84	0.035	-2.93126
62	0	6.248	0.038	-3.07582
55	0	4.84	0.011	-3.15494

53	0	4.72	0.007	-3.17752
25	1	7.19	0.034	-3.22853
68	0	7.873	0.03	-3.34949
65	0	7.476	0.014	-3.44997
72	0	9.959	0.039	-3.52121
61	0	10.61	0.039	-3.60098
54	0	13.7	0.068	-3.70932
59	0	10.299	0.02	-3.73995
60	0	9.778	0.004	-3.82524
66	0	14.495	0.035	-4.11429
56	0	17.55	0.032	-4.51658
67	0	23.862	0.011	-5.48572
58	0	24.82	0.007	-5.64038
74	0	31.43	0.015	-6.37575

*patients' ID numbers as in Table S3 and S4

(b)

patient ID no.	miR-let-7b expression	miR-155 expression	prediction index	predicted as malignant (1)	true malignant (1)
31	2.07	26.04	239.7769	1	1
34	1.57	6.49	57.63054	1	1
50	2.53	6.42	56.8605	1	1
51	2.65	6.12	54.04978	1	1
49	3.99	5.92	52.02157	1	1
32	2.57	5.43	47.62872	1	1
46	3.93	5.13	44.66606	1	1
28	3.4	4.8	41.65537	1	1
48	1.38	3.31	28.01596	1	1
47	1.18	2.54	20.86401	1	1
27	1.59	1.8	13.91691	1	1
52	2.06	1.76	13.48652	1	1
33	1.23	1.08	7.250561	1	1
26	1.29	1.01	6.590804	1	1
30	2.8	0.66	3.143754	1	1
29	2.53	0.5	1.685625	1	1
79	3.46	0.072	-2.41732	0	0
45	1.62	0.0472	-2.42301	0	1
87	6.46	0.042	-3.06452	0	0
77	4.23	0.008	-3.10816	0	0
91	4.34	0.005	-3.1496	0	0
92	4.37	0.005	-3.15327	0	0
78	5.06	0.008	-3.20986	0	0
80	5.88	0.018	-3.21713	0	0
90	5.71	0.011	-3.26154	0	0
81	6.12	0.013	-3.29314	0	0
88	5.81	0.008	-3.30176	0	0
94	5.28	0.001	-3.30205	0	0
76	5.84	0.007	-3.31475	0	0
93	6.45	0.006	-3.39882	0	0
89	7.3	0.005	-3.51229	0	0
82	7.55	0.004	-3.55224	0	0
86	10.53	0.002	-3.93602	0	0
85	14.09	0.004	-4.35359	0	0

*patients' ID numbers as in Table S3 and S4
