# Supplementary material for: Tumor and Cerebrospinal Fluid microRNAs in Primary Central Nervous System Lymphomas
Source: Cancers (Basel). 2019 Oct 25;11(11):1647. doi: 10.3390/cancers11111647 (PMC6895823; doi:10.3390/cancers11111647)
Supplement: Supplementary file 1 [file cancers-11-01647-s001.zip › Suppl ZIP_PROOFS/Table_S_2.pdf]

Table S2. Patients with non-neoplastic cerebral tumors (n=23): clinical diagnosis and miRNA expression level in the cerebrospinal fluid (CSF).

pt. ID no.	age/sex	miRNA from CSF			Baraniskin's diagnostic algorithim[1]: positive for lymphoma (1), negative for lymphoma (2)	FCM diagnosis positive for lymphoma (1), negative for lymphoma (2)	Cytology	ICD-10
		miR-19b-1	miR-21	miR-92a				
95	36/m	0.612	9.792	1.29	2	2	L	G37.9
96	49/f	0.565	4.641	0.52	2	2	L, M, E	D33.1
97	54/f	2.481	75.747	2.80	1	2	L, M, E, Eo	G04.9 (viral)
98	38/m	0.484	1.191	0.38	2	2	L, M, E	I67.7
99	17/m	1.131	14.654	2.31	2	2	D	G04.8 (HD)
100	61/f	0.381	3.966	0.66	2	2	L, M	I67.7
101	48/m	1.083	36.305	2.92	2	2	L, M*	G04.9 (ADEM)
102	30/f	1.316	9.913	2.09	2	2	L, M, Neu*	G35 (SM)
103	48 /f	1.988	11.406	2.25	1	2	L, M*	G37.9
104	59/m	1.474	5.381	1.63	2	2	L*	G04.8
105	81/f	1.359	6.173	2.51	2	2	L, M	D43.1 (meningioma)
106	31/f	1.276	4.960	1.35	2	2	L, M	D33
107	9/f	31.89	0.500	1.62	2	2	L, M	G40.2
108	13/f	30.08	1.835	2.08	2	2	L, E, D	I87.3
109	40/m	29.94	1.800	2.51	2	2	L, M	G37.9
110	26/m	1.526	13.289	1.88	1	2	L, M	D33.1
111	14/m	0.749	16.43	1.39	2	2	L, M	G35 (SM)
112	16/f	0.904	7.435	1.47	2	2	L, M	G35 (SM)
113	12/m	0.924	11.35	2.31	2	2	L, M	G35 (SM)
114	17/f	0.663	12.07	0.98	2	2	L, M*	G36 [Devic]
115	14/m	1.148	3.515	1.27	2	2	L, M	G35 (SM)?
116	16/f	3.696	1.933	3.26	2	2	E, D	G35 (SM)
117	70/f	3.431	65.979	45.64	1	2	L, D*	G04.9 (no viral)

[1]Baraniskin, A.; Kuhnhenn, J.; Schlegel, U.; Chan, A.; Deckert, M.; Gold, R.; Maghnouj, A.; Zollner, H.; Reinacher-Schick, A.; Schmiegel, W.; Hahn, S. A.; Schroers, R., Identification of microRNAs in the cerebrospinal fluid as marker for primary diffuse large B-cell lymphoma of the central nervous system. Blood 2011, 117, (11), 3140-6.

age/sex, m-male, f-female; miRNA level expression (miR-19b-1, miR-21, miR-92a) from CSF; Baraniskin's algorithim positive for lymphoma (19b-1 ≥1,4 and miR-21 ≥8 or miR-92a ≥2,5) (1), negative for lymphoma diagnosis (lower value of the above-mentioned miRNAs) (2); cytological smears: L – lymphocytes, M – macrophages, D – debris, E – erythrocytes, Eo – eosinophils, Neu – neutrophils), *Cerebrospinal fluid pleocytosis level (Lymphocytic pleocytosis?); Clinical diagnosis of non-neoplastic CNS tumors according to the International Classification of Diseases, Tenth Revision, Clinical Modification (ICD-10-CM).

Diseases of the nervous system codes:

C71 Malignant neoplasm of cerebrum, except lobes and ventricles,

C71.9 Malignant neoplasm of brain, unspecified,

D33 Benign neoplasm of brain and other parts of central nervous system,
D33.1 Benign neoplasm of brain, infratentorial,
D33.2 Benign neoplasm of brain, unspecified,
D43.1 Neoplasm of uncertain behavior of brain, infratentorial,
G04.8 Other encephalitis, myelitis and encephalomyelitis,

- HD, Huntington's disease.

G04.9 Encephalitis, myelitis and encephalomyelitis, unspecified,

- ADEM, Acute Disseminated Encephalomyelitis.

G35 Multiple sclerosis (SM),
G36 Neuromyslitis optica [Devic],
G37.9 Demyelinating disease of central nervous system, unspecified,
G40.2 Localization-related (focal) (partial) symptomatic epilepsy and epileptic syndromes with complex partial seizures,
I67.7 Central nervous system vasculitis (cerebral NEC),
I87.3 Cerebral venous thrombosis CVT in the puerperium.
