# Supplementary material for: Tumor and Cerebrospinal Fluid microRNAs in Primary Central Nervous System Lymphomas
Source: Cancers (Basel). 2019 Oct 25;11(11):1647. doi: 10.3390/cancers11111647 (PMC6895823; doi:10.3390/cancers11111647)
Supplement: Supplementary file 1 [file cancers-11-01647-s001.zip › Suppl ZIP_PROOFS/Table_S_3.pdf]

Table S3. Clinical and pathomorphological characteristics of CNS DLBCL patients (n=52), along with miRNA expression levels in tumor biopsy samples.

pt. ID no.*	age/ sex	Histo-pathology	IHC		miRNA extracted from FFPET					Prognostic Score System for Primary CNS Lymphomas		Treatment			Response Status/Last FU (mo)
			cell of origin: GCB ABC CD5(+)	BCL2: (++)h (+) (-)	let-7b	miR-125b	miR-196b	miR-155	miR-9	IELSG: 0-1=0 2-3=1 4-5=2	MSKCC: 1=<50y 2=>50y >70KFS 3=>50y <70KFS	HD-MTX	RTH	ASCT	ANED or DOD
1	45/f	CNS DLBCL	GCB	(++)h	1.94	1.43	0.01	1.71	0.45	1	1	yes	no	no	DOD (4)
2	82/m	CNS DLBCL	ABC	(++)h	0.72	3.28	0.04	3.48	3.21	2	3	no	yes	no	ANED (39)
3	59/m	CNS DLBCL	ABC	(+)	5.54	1.34	0.01	2.05	0.34	1	2	yes	no	yes	ANED (31)
4	40/f	CNS DLBCL	CD5(+)	(-)	2.63	3.01	0.09	5.38	0.40	2	1	yes	no	no	DOD (3)
5	55/m	CNS DLBCL	ABC	(++)h	4.18	4.32	0.001	1.077	2.421	1	2	yes	no	yes	DOD (6)
6	63/f	CNS DLBCL	ABC	(++)h	1.05	2.38	0.003	1.243	1.265	1	2	yes	yes	no	ANED (45)
7	66/f	CNS DLBCL	CD5(+)	(++)h	3.13	4.23	0.001	1.007	0.071	1	2	yes	no	no	ANED (50)
8	36/m	CNS DLBCL	GCB	(++)h	1.40	0.63	0.045	3.007	0.248	2	1	yes	yes	no	DOD (6)
9	47/m	CNS DLBCL	GCB	(++)h	4.17	1.74	0.005	15.103	0.154	1	1	yes	no	yes	ANED (34)
10	54/m	CNS DLBCL	ABC	(+)	2.30	1.14	0.088	10.406	0.620	2	3	yes	yes	no	DOD (10)
11	54/f	CNS DLBCL	ABC	(+)	6.52	4.21	0.005	19.136	3.107	1	2	yes	yes	no	ANED (60)
12	66/f	CNS DLBCL	CD5(+)	(++)h	0.44	0.28	0.012	1.943	0.093	2	3	yes	yes	no	DOD (86)
13	50/m	CNS DLBCL	CD5(+)	(+)	1.59	1.74	0.051	0.510	0.959	1	1	yes	no	no	ANED (39)
14	70/f	CNS DLBCL	ABC	(+)	1.98	0.44	0.158	53.291	0.305	2	3	yes	yes	no	DOD (7)
15	73/f	CNS DLBCL	ABC	(+)	3.20	2.05	0.002	22.246	0.557	2	3	yes	no	no	DOD (2)

16	50/m	CNS DLBCL	CD5(+)	(+)	3.57	0.07	5.735	25.700	0.364	1	1	yes	no	no	DOD (29)
17	62/f	CNS DLBCL	CD5(+)	(+)	0.57	0.47	0.042	3.110	0.387	1	2	yes	no	no	DOD (46)
18	57/f	CNS DLBCL	ABC	(+)	1.22	1.13	0.046	3.997	0.435	0	3	yes	yes	no	DOD (59)
19	51/f	CNS DLBCL	ABC	(+)	0.89	0.65	0.129	11.874	1.341	1	2	yes	yes	no	ANED (76)
20	62/m	CNS DLBCL	ABC	(+)	1.59	1.57	0.020	2.619	0.090	2	3	no	no	no	DOD (1)
21	59/f	CNS DLBCL	ABC	(+)	3.83	7.97	0.083	5.224	2.962	1	2	yes	yes	no	ANED (57)
22	65/f	CNS DLBCL	ABC	(+)	3.19	2.20	0.051	3.395	0.011	2	2	no	yes	no	DOD (22)
23	67/m	CNS DLBCL	ABC	(+)	6.23	5.89	0.005	0.534	3.861	2	2	yes	yes	no	ANED (21)
24	63/f	CNS DLBCL	CD5(+)	(+)	7.05	4.58	0.015	1.168	0.832	1	2	yes	yes	no	ANED (54)
25	42/m	CNS DLBCL	ABC	(+)	7.19	11.71	0.003	0.034	5.752	1	1	yes	no	yes	ANED (54)
26	49/f	CNS DLBCL	ABC	(+)	1.29	1.32	0.007	1.01	0.41	1	1	yes	yes	no	ANED (68)
27	65/m	CNS DLBCL	ABC	(++)h	1.59	0.83	0.002	1.80	0.79	2	3	no	no	no	DOD (2)
28	49/f	CNS DLBCL	ABC	(++)h	3.40	3.75	0.017	4.80	2.41	2	1	yes	no	no	DOD (40)
29	66/f	CNS DLBCL	ABC	(+)	2.53	3.99	0.0005	0.50	2.81	2	2	yes	yes	no	ANED (29)
30	74/f	CNS DLBCL	ABC	(++)h	2.80	3.70	0.057	0.66	2.21	1	2	yes	yes	no	DOD (32)
31	59/m	CNS DLBCL	ABC	(+)	2.07	0.34	0.079	26.04	0.21	1	2	yes	no	yes	ANED (25)
32	68/f	CNS DLBCL	ABC	(++)h	2.57	3.65	0.082	5.43	2.43	2	2	yes	yes	no	ANED (26)
33	31/f	CNS DLBCL	GCB	(+)	1.23	0.85	0.097	1.08	0.26	1	1	yes	yes	no	DOD (10)
34	64/m	CNS DLBCL	ABC	(+)	1.57	2.47	0.189	6.49	2.29	1	3	yes	yes	no	DOD (14)
35	81/f	CNS DLBCL	ABC	(++)h	1.92	1.53	0.06	2.43	0.86						
36	77/f	CNS DLBCL	CD5(+)	(++)h	1.35	0.52	0.46	13.12	0.06						
37	59/m	CNS DLBCL	ABC	(+)	2.53	1.61	0.241	5.938	0.444						
38	52/m	CNS DLBCL	ABC	(+)	1.57	0.86	0.101	23.720	0.446						

39	62/f	CNS DLBCL	CD5(+)	(+)	7.88	6.37	0.128	18.993	1.062
40	68/f	CNS DLBCL	ABC	(+)	2.22	1.24	0.011	2.512	0.130
41	67/f	CNS DLBCL	ABC	(+)	20.45	19.70	0.012	0.555	1.724
42	67/m	CNS DLBCL	GCB	(+)	3.37	0.53	0.086	0.529	0.253
43	73/f	CNS DLBCL	CD5(+)	(++)h	0.75	0.79	0.000	4.661	0.011
44	66/m	CNS DLBCL	CD5(+)	(++)h	3.09	3.04	0.001	8.365	1.353
45	55/f	CNS DLBCL	ABC	(+)	1.62	1.37	0.029	0.0472	0.001
46	79/f	CNS DLBCL	ABC	(+)	3.93	3.44	0.001	5.13	1.51
47	58/f	CNS DLBCL	ABC	(+)	1.18	1.02	0.047	2.54	0.34
48	40/f	CNS DLBCL	ABC	(++)h	1.38	1.30	0.0031	3.31	1.42
49	69/f	CNS DLBCL	CD5(+)	(++)h	3.99	5.46	0.142	5.92	1.85
50	50/m	CNS DLBCL	ABC	(++)h	2.53	1.07	0.183	6.42	0.72
51	52/f	CNS DLBCL	ABC	(+)	2.65	3.14	0.002	6.12	1.92
52	67/m	CNS DLBCL	ABC	(+)	2.06	1.46	0.174	1.76	1.04

*patients no. 1-34 with clinical data, patients no. 35-52 without clinical data.
age/sex., m-male, f-female; Histopathology, primary histopathological diagnosis from CNS tumor, IHC., immunohistochemical examination, cell of origin: GCB - germinal center B-cell type, ABC - activated B-cell type (non-GCB), CD5(+) CD5 – positive immunohistochemical subgroup; BCL2., BCL2 staining: (++)h, higher BCL2 staining compared to T lymphocytes, (+) BCL2 staining similar or weaker compared to T lymphocytes, (-) lack of BCL2 staining on neoplastic cells; miRNA level expression extracted from FFPET (formalin fixed paraffin embedded tissue);
Prognostic Scoring System for Primary CNS Lymphomas:
1) IELSG (the International Extranodal Lymphoma Study Group)
prognostic score is based on age over 60 years, performance status (PS) more than 1, elevated lactate dehydrogenase (LDH) serum level, high CSF protein concentration, and involvement of deep regions of the brain. These five variables are used to design a prognostic score (0-5). Each variable was assigned a value of either 0, if favorable, or 1, if unfavorable. 2) MSKCC (the Memorial Sloan-Kettering Cancer Center Prognostic Model) identified three distinct prognostic classes: class 1 (patients <50 years), class 2 (patients > or =50; Karnofsky performance score [KPS] > or =70) and class 3 (patients > or =50; KPS <70). These three classes significantly distinguished outcome with regard to both overall and failure-free survival; Treatment.: HD-MTX, high-dose metotrexate, RTH, radiotherapy, ASCT, autologous stem cell transplantation; Response Status: ANED, Alive, no evidence of disease; DOD, Died of Disease progression / Last FU (follow up) (mo).
