# Supplementary material for: Tumor and Cerebrospinal Fluid microRNAs in Primary Central Nervous System Lymphomas
Source: Cancers (Basel). 2019 Oct 25;11(11):1647. doi: 10.3390/cancers11111647 (PMC6895823; doi:10.3390/cancers11111647)
Supplement: Supplementary file 1 [file cancers-11-01647-s001.zip › Suppl ZIP_PROOFS/Table_S_4.pdf]

Table S4. Non-neoplastic cerebral or cerebellum tumor patients (n=42): pathomorphological diagnosis and tumor miRNA expression levels.

pt. ID no.	age/sex	HP, non-neoplastic cerebral hemispheres (1) or cerebellum (2) tumors	miRNA extracted from FFPET				
			let-7b	miR-125b	miR-196b	miR-155	miR-9
53	33/m	reaction process, gliosis (1)	4.72	11.33	0.0003	0.007	6.50
54	25/m	cavernous malformation, gliosis (1)	13.70	47.72	0.0170	0.068	0.89
55	59/m	reaction process, gliosis (1)	4.84	8.54	0.0003	0.011	3.07
56	68/m	cerebral hemispheres tissue (1)	17.55	15.79	0.0197	0.032	1.03
57	26/m	reaction process, gliosis (1)	4.84	8.47	0.0023	0.035	1.15
58	63/m	reaction process, gliosis (2)	24.82	13.10	0.0009	0.007	5.47
59	64/m	cavernous malformation (2)	10.299	5.738	0.0029	0.020	6.066
60	28/f	cyst (2)	9.778	5.145	0.0004	0.004	7.151
61	32/m	abscess, reaction process (1)	10.610	6.699	0.0005	0.039	1.201
62	43/f	cavernous malformation, gliosis (1)	6.248	3.951	0.0022	0.038	0.502
63	47/f	cavernous malformation, gliosis (1)	4.459	2.531	0.0046	0.064	0.371
64	41/f	hematoma, reaction process (1)	1.588	1.248	0.0016	0.132	0.840
65	34/f	cavernous malformation, gliosis (1)	7.476	4.971	0.0001	0.014	0.831
66	52/m	gliosis (1)	14.495	17.187	0.0008	0.035	2.097
67	76/m	hematoma (1)	23.862	29.304	0.0025	0.011	5.822
68	40/m	gliosis (1)	7.873	11.065	0.0001	0.030	3.608
69	50/m	ischemic necrosis, gliosis (1)	5.621	11.273	0.0002	0.078	0.648
70	51/m	cavernous malformation, gliosis (1)	3.185	0.594	0.0357	0.041	0.011
71	47/m	hematoma (1)	3.724	3.878	0.001	0.125	0.090
72	78/f	cerebral hemisphere tissue (1)	9.959	18.410	0.0005	0.039	5.500
73	65/m	reaction process (1)	3.874	5.783	0.0003	0.382	3.297
74	18/f	cerebellum tissue (2)	31.430	42.907	0.0008	0.015	12.135
75	69/f	reaction process, lymphocytic inflatation (1)	27.868	5.652	0.100	0.615	4.079

76	58/f	reaction process (1)	5.84	10.76	0.0007	0.007	11.64
77	73/m	cavernous malformation, gliosis (1)	4.23	10.42	0.0009	0.008	11.52
78	62/m	hematoma (1)	5.06	9.47	0.0008	0.008	11.33
79	56/f	reaction process, toxoplasmosis? gliosis (1)	3.46	2.62	0.0018	0.072	2.09
80	30/f	ischemic necrosis, gliosis (1)	5.88	11.48	0.0006	0.018	10.62
81	54/f	hematoma (1)	6.12	13.96	0.0007	0.013	10.08
82	35/f	reaction process, gliosis (1)	7.55	17.22	0.0003	0.004	24.02
83	34/m	focal cortical dysplasia (FCD) type IIA (1), ischemic necrosis (1)	6.54	10.50	0.0002	0.008	9.88
84	57/m	gliosis (1), brain tissue (1)	5.88	11.45	0.0006	0.008	10.83
85	67/f	cerebral hemispheres tissue (1)	14.09	9.44	0.0011	0.004	3.25
86	65/f	hematoma, gliosis (1)	10.53	1.75	0.17	0.002	0.02
87	27/m	cavernous malformation, gliosis (1)	6.46	6.90	0.0021	0.042	2.62
88	28/f	focal cortical dysplasia (FCD) type IIB (1)	5.81	10.56	0.0015	0.008	8.45
89	34/f	cerebellum tissue (2)	7.30	7.32	0.0007	0.005	11.63
90	31/m	reaction process (1)	5.71	14.41	0.0019	0.011	12.17
91	32/f	ischemic necrosis (1)	4.34	9.68	0.0002	0.005	14.43
92	42/m	cerebral hemispheres tissue (1)	4.37	7.99	0.0006	0.005	9.57
93	36/m	hematoma (1)	6.45	6.57	0.0010	0.006	7.75
94	54/m	cerebral hemispheres tissue (1)	5.28	5.94	0.0017	0.001	4.03

age/sex, m-male, f-female; HP. histopathological diagnosis from non-neoplastic CNS tumor; tumor miRNA expression levels (RNA extracted from formalin fixed paraffin embedded tissue).
