# Supplementary material for: Tumor and Cerebrospinal Fluid microRNAs in Primary Central Nervous System Lymphomas
Source: Cancers (Basel). 2019 Oct 25;11(11):1647. doi: 10.3390/cancers11111647 (PMC6895823; doi:10.3390/cancers11111647)
Supplement: Supplementary file 1 [file cancers-11-01647-s001.zip › Suppl ZIP_PROOFS/Table_S_5.pdf]

Table S5. Patients with CNS DLBCL (n=30), clinical diagnosis and miRNA expression level in the cerebrospinal fluid (CSF).

no.	age/ sex	Histopathology	FCM diagnosis: positive for lymphoma (1), negative for lymphoma (2)	Cytology	miRNA from CSF							
					miR-19b	miR-21	miR-92a	miR-9	miR-9*	miR-155	miR-125b	miR-196b
19	50/m	CNS DLBCL	2	L, M, E, D	5.897	8.388	11.162	0.10	0.58			
118	64/m	CNS DLBCL	1 (minimal involvement)	L, M, E	1.744	26.308	2.249	2.19	1.08			
119	69/m	CNS DLBCL	2	L, M, E	3.929	18.105	3.524			0.08		
24	62/f	CNS DLBCL	2	L, M, E	0.966	3.546	1.345					
29	67/m	CNS DLBCL	2	L*	9.448	93.073	23.730			0.25		
28	51/f	CNS DLBCL	2	L	2.236	11.390	4.570	0.37	0.33			
44	49/f	CNS DLBCL	1 (minimal involvement)	L*	7.463	38.362	19.483	0.07	0.19			
120	20/m	CNS DLBCL	2	L, D	1.012	17.655	2.862	0.04				
30	61/m	CNS DLBCL	2	L, D	91.136	520.987	78.325			0.01		
31	59/f	CNS DLBCL	2	L, M	0.652	67.028	6.724	0.63				
121	73/f	CNS DLBCL	1 (minimal involvement)	L, M*	6.323	33.581	8.412	0.02	1.46	0.30		
33	65/f	CNS DLBCL	1 (minimal involvement)	L, M	1.443	24.959	2.131	0.00	0.18			
122	28/m	CNS DLBCL	1 (minimal involvement)	L*	1.040	48.824	1.636					
38	42/m	CNS DLBCL	2	L* M	6.471	3.564	5.931				2.93	0.13
123	60/f	CNS DLBCL	2	L, M, E	1.392	7.985	3.532				6.40	0.12
124	50/m	CNS DLBCL	2	L, M, E	1.236	9.913	1.973				5.09	0.00

125	56/f	CNS DLBCL	2	L, M, E	14.023	14.036	14.016	0.07	0.48		3.99	0.03
37	63/f	CNS DLBCL	2	L, M	7.918	20.676	11.603			3.89	1.85	0.00
126	68/f	CNS DLBCL	2	L* M	1.773	11.068	2.093		0.23	0.24	0.81	0.05
9	67/f	CNS DLBCL	2	L, M, D	0.879	7.354	1.891				5.60	0.00
1	45/f	CNS DLBCL	2	L, M	1.872	3.084	2.779			0.00	13.99	0.01
10	36/m	CNS DLBCL	2	L, M, E, D	9.046	11.838	14.698	0.00		0.02		
127	51/m	CNS DLBCL	2	L, M, E, D	1.086	2.918	1.315					
128	59/f	CNS DLBCL	1 (minimal involvement)	L, M, D	1.678	13.419	1.242	0.00		0.03		
129	64/m	CNS DLBCL	1 (massive involvement)	L, M, E	2.639	80.651	2.184	0.01		0.02		
7	52/m	CNS DLBCL	2	L, M	0.493	7.228	1.460					
130	52/m	CNS DLBCL	2	L, M	1.158	11.509	2.656					
5	59/m	CNS DLBCL	2	L, M	1.318	7.983	2.043					
131	79/f	CNS DLBCL	2	L, M, E	3.906	93.414	22.877					
8	63/f	CNS DLBCL	1 (minimal involvement)	L, M	1.305	9.893	2.362					

patients number 118-131 with CNS DLBCL with no simultaneous analysis of miRNA in FFPET (formalin fixed paraffin embedded tissue), patients no. 1, 5, 7-10, 19, 24, 28-31, 33, 37-38 with simultaneously analyzed miRNA in FFPET and CSF, age/sex., m-male, f-female; Histopathology, primary histopathological diagnosis based on CNS tumor; Cytological smears: L – lymphocytes, M – macrophages, D – debris, E – erythrocytes; miRNA from CSF, miRNA level expression (miR-19b, miR-21, miR-92a, miR-9, miR-9*, miR-155, miR-125b, miR-196b) from CSF.
