# Supplementary material for: Tumor and Cerebrospinal Fluid microRNAs in Primary Central Nervous System Lymphomas
Source: Cancers (Basel). 2019 Oct 25;11(11):1647. doi: 10.3390/cancers11111647 (PMC6895823; doi:10.3390/cancers11111647)
Supplement: Supplementary file 1 [file cancers-11-01647-s001.zip › Suppl ZIP_PROOFS/Table_S_6.pdf]

Table S6. The clones of Dako antibodies used for immunohistochemistry analyses.

**MoAb**	**CD3**	**CD5**	**CD10**	**CD20**	**BCL2**	**BCL6**	**MUM1**	**Ki-67**
Clone	F7.2.38	SP19	56C6	L26	124	PG-B6p	MUM1p	MIB-1
Dilution	1:50	F	F	F	F	F	F	F

MoAb, monoclonal antibody; Dako, (Glostrup, Denmark); F - FLEX, EnVision FLEX. High-sensitivity visualization system, intended for use in immunohistochemistry together with Dako Omnis autostainer was used.
