# Supplementary material for: Tumor and Cerebrospinal Fluid microRNAs in Primary Central Nervous System Lymphomas
Source: Cancers (Basel). 2019 Oct 25;11(11):1647. doi: 10.3390/cancers11111647 (PMC6895823; doi:10.3390/cancers11111647)
Supplement: Supplementary file 1 [file cancers-11-01647-s001.zip › Suppl ZIP_PROOFS/Table_S_7.pdf]

Table S7. Antibodies used for flow cytometry.

**MoAb**	Clone	Source
**CD45**	2D1	
		Becton Dickinson
**HLA-DR**	L243	
**CD19**	4G7	
**CD20**	L27	
**CD3**	SK7	
**CD4**	SK3	
**CD5**	L17F12	
**CD8**	SK1	
**CD10**	HI10a	
**CD11c**	S-HCL-3	
**CD71**	L01.1	
**κ/λ**	TB28-2/1-155-2	
**CD43**	DFT-1	Serotec

MoAb, monoclonal antibodies; κ/λ (kappa/lambda)
