# Supplementary material for: Tumor and Cerebrospinal Fluid microRNAs in Primary Central Nervous System Lymphomas
Source: Cancers (Basel). 2019 Oct 25;11(11):1647. doi: 10.3390/cancers11111647 (PMC6895823; doi:10.3390/cancers11111647)
Supplement: Supplementary file 1 [file cancers-11-01647-s001.zip › Suppl ZIP_PROOFS/Table_S_8.pdf]

Table S8. MicroRNA sequences with GC content.

miRBase ID	TaqMan ID	Sequence	Length (nt)	GC (nt)	GC (%)
miR-9-5p	ID 000583	UCUUUGGUUAUCUAGCUGUAUGA	23	8	34,78
miR-9-3p*	ID 002231	AUAAAGCUAGAUAACCGAAAGU	22	7	31,82
miR-let-7b-5p	ID 000378	UGAGGUAGUAGGUUGUGUGGUU	22	10	45,46
miR-15b-5p	ID 000390	UAGCAGCACAUCAUGGUUUACA	22	9	40,91
miR-19b-3p	ID 000396	UGUGCAAAUCCAUGCAAAACUGA	23	9	39,13
miR-20b-5p	ID001014	CAAAGUGCUCAUAGUGCAGGUAG	23	11	47,83
miR-21-5p	ID 000397	UAGCUUAUCAGACUGAUGUUGA	22	8	36,36
miR-29a-5p	ID 002447	ACUGAUUUCUUUUGGUGUUCAG	22	8	36,36
miR-29b-1-5p	ID 002165	GCUGGUUUCAUAUGGUGGUUUAGA	24	10	41,67
miR-29b-2-5p	ID 002166	CUGGUUUCACAUGGUGGCUUAG	22	11	50
miR-92a-3p	ID 000431	UAUUGCACUUGUCCCGGCCUGU	22	12	54,55
miR-125b-5p	ID 000449	UCCCUGAGACCCUAACUUGUGA	22	11	50
miR-155-5p	ID 000479	UUAAUGCUAAUCGUGAUAGGGG	22	9	40,91
miR-196b-5p	ID 002215	UAGGUAGUUUCCUGUUGUUGGG	22	10	45,46
miR-24-3p	ID 000402	UGGCUCAGUUCAGCAGGAACAG	22	12	54,55
